# Antibodies from serum and CSF of multiple sclerosis patients bind to oligodendroglial and neuronal cell-lines

**DOI:** 10.1093/braincomms/fcad164

**Published:** 2023-05-23

**Authors:** Faisal Hayat Nazir, Anna Wiberg, Malin Müller, Sara Mangsbo, Joachim Burman

**Affiliations:** Department of Medical Sciences, Neurology, Uppsala University, Uppsala SE-751 85, Sweden; Department of Medical Sciences, Neurology, Uppsala University, Uppsala SE-751 85, Sweden; Department of Medical Sciences, Neurology, Uppsala University, Uppsala SE-751 85, Sweden; Department of Pharmacy, Science for Life Laboratory, Uppsala University, Uppsala SE-751 23, Sweden; Department of Medical Sciences, Neurology, Uppsala University, Uppsala SE-751 85, Sweden

**Keywords:** immunoglobulins, multiple sclerosis, cell-based ELISA, antibodies, cell-lines

## Abstract

Multiple sclerosis is a highly complex and heterogeneous disease. At the onset it often presents as a clinically isolated syndrome. Thereafter relapses are followed by periods of remissions, but eventually, most patients develop secondary progressive multiple sclerosis. It is widely accepted that autoantibodies are important to the pathogenesis of multiple sclerosis, but hitherto it has been difficult to identify the target of such autoantibodies. As an alternative strategy, cell-based methods of detecting autoantibodies have been developed. The objective of this study was to explore differences in the binding of antibodies from sera and CSF of multiple sclerosis patients and controls to oligodendroglial and neuronal cell-lines, related to antibody type, immunoglobulin (IgG/IgM), matrix (serum/CSF) and disease course. The oligodendroglial and neuronal cell-lines were expanded in tissue culture flasks and transferred to 96-well plates at a concentration of 50 000 cells/well followed by fixation and blocking with bovine serum albumin. Sera and CSF samples, from healthy controls and multiple sclerosis patients, were incubated with the fixed cells. Epitope binding of immunoglobulins (IgG and IgM) in sera and CSF was detected using biotinylated anti-human IgM and IgG followed by avidin conjugated to horseradish peroxidase. Horseradish peroxidase activity was detected with 3,3′,5,5′-tetramethylbenzidine substrate. Serum from 76 patients and 30 controls as well as CSF from 62 patients and 32 controls were investigated in the study. The binding was similar between clinically isolated syndrome patients and controls, whereas the largest differences were observed between secondary progressive multiple sclerosis patients and controls. Antibodies from multiple sclerosis patients (all disease course combined) bound more to all investigated cell-lines, irrespectively of matrix type, but binding of immunoglobulin G from CSF to human oligodendroglioma cell-line discriminated best between multiple sclerosis patients and controls with a sensitivity of 93% and a specificity of 96%. The cell-based enzyme linked immunosorbent assay (ELISA) was able to discriminate between multiple sclerosis patients and controls with a high degree of accuracy. The disease course was the major determinant for the antibody binding.

## Teaser

Nazir *et al*. measured antibody binding to neuronal and oligodendroglial cell-lines with a cell-based ELISA. They report that this method could discriminate between multiple sclerosis patients and healthy controls with a high degree of accuracy. The disease course was the major determinant for the antibody binding.

## Graphical Abstract

**Graphical Abstract:**
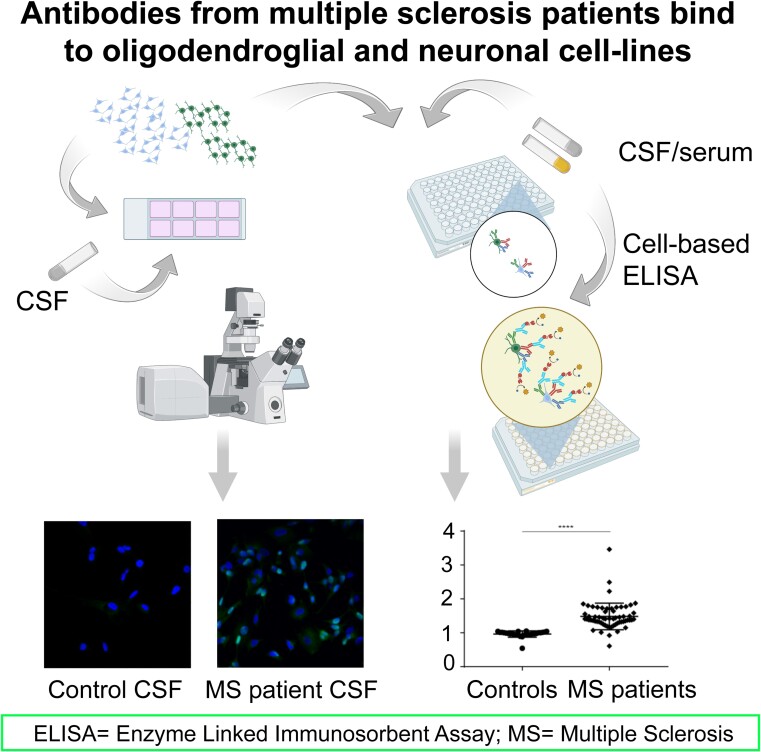

## Introduction

Multiple sclerosis (MS) is an autoimmune, inflammatory and demyelinating disease of the central nervous system. It is estimated that 2.8 million people are living with MS worldwide. Although the precise aetiology remains unclear, there are both genetic and environmental risk factors. Smoking, low levels of vitamin D, certain viral infections, and obesity are some of the confirmed causatives.^1^ Over 200 genes have been identified affecting the risk of developing MS, with the human leukocyte antigen Class II gene as the main susceptible allele.^2^

The International Advisory Committee on Clinical Trials of MS in 2013 defined MS into four basic MS disease courses (also known as phenotypes of MS): clinically isolated syndrome (CIS), relapsing remitting MS (RRMS), secondary progressive MS (SPMS) and primary progressive MS (PPMS).^3^ CIS is the first clinical presentation of a disease that shows characteristics of inflammatory demyelination that could be MS; however, it still has to fulfil the criteria of dissemination in time.^4^ Individuals with CIS may or may not go on to develop relapsing-remitting MS (RRMS). RRMS is the most common type of MS at onset, accounting for around 85% of all patients. It involves spontaneous relapses or worsening of neurologic function followed by periods of remission or recovery where symptoms improve or resolve completely.^5^ Individuals with RRMS may gradually transit to SPMS, characterized by neurodegeneration, central nervous system atrophy and accumulation of disability. Individuals with disease progression from the onset are diagnosed with PPMS.^5^

Several studies indicate that autoantibodies are central to the pathogenesis of MS and related disorders.^6,7^ For a long time neuromyelitis optica (NMO) was considered a variant of MS.^8^*Lennon et al*., described a novel serum antibody (NMO-IgG) that bound specifically to aquaporin-4 expressed on astrocytes. This antibody was often found in patients with NMO, but very rarely in patients with other conditions (e.g. MS) and it was concluded that NMO-IgG is a specific and diagnostic marker autoantibody of NMO.^9–11^ Furthermore, myelin oligodendrocyte glycoprotein (MOG) autoantibodies have been extensively studied in experimental models of MS. However, attempts to translate these findings into human disease have yielded controversial results, especially with regard to MOG antibodies as a prognostic biomarker in MS.^11,12^ The presence of MOG antibodies confirmed in a subset of predominantly paediatric patients with varying symptomatology, collectively referred to as MOG-associated diseases (MOGAD).^11–13^

In an extensive study to identify cells to which potential autoantibodies could bind, Lily *et al.* investigated several cancerous cell-lines of oligodendrocytic, astrocytic, neuronal, striated muscle and endothelial origin.^14^ Using flow cytometry, they identified neuronal and oligodendrocyte precursor cell-lines with high antibody binding potential for sera from MS patients.^14^ Building on their experiences, we used a similar set-up of neuronal and oligodendrocyte precursor cell-lines to detect potential antibodies in sera and CSF from MS patients with different disease courses (CIS, RRMS, SPMS) using a cell-based enzyme linked immunosorbent assay (ELISA).

## Materials and methods

### Ethical statement

The study was approved by the Ethical Review Board in Uppsala (Dnr 2008/182). All patients provided written informed consent.

### Patients

The patients were recruited from the outpatient clinic at Uppsala University Hospital, Uppsala, Sweden. Blood samples from 30 healthy controls, 9 CIS, 45 RRMS and 25 SPMS patients as well as CSF samples from 32 healthy controls, 8 CIS, 30 RRMS and 19 SPMS patients were used in the study. Blood samples and CSF samples were only partially overlapping. RRMS and SPMS patients were diagnosed with 2010 revised McDonald criteria for MS diagnosis.^15^ SPMS patients were clinically deteriorating in the absence of relapses. The duration of disease was calculated from the date of the first symptom presentation. Disability was scored with the expanded disability status scale. Demographic data and clinical characteristics are summarized in Supplementary Table 1 for serum samples and Supplementary Table 2 for CSF samples. A more detailed description of the patients is provided in Supplementary Table 3.

### Sample collection

Blood samples were collected by venipuncture. Following centrifugation, heparin plasma was collected and snap-frozen at −80°C. Following lumbar puncture, CSF was collected in a polypropylene tube and centrifuged at 250 × *g* for 5 min. The supernatant was pipetted off, gently mixed, and aliquoted in polypropylene tubes stored at −80°C.

### Cell-lines

Three cell-lines were used in this study: the human oligodendroglioma cell-line (HOG), derived from human oligodendroglioma; the human oligodendrocytic glial cell-line (MO3.13), created by fusing human rhabdomyosarcoma with adult human oligodendrocytes; and the neuronal cell-line (SK-N-SH), derived from human neuroblastoma cells. The HOG and MO3.13 cell-lines were provided by Dr Roumen Balabanov, Department of Neurological Sciences, Multiple Sclerosis Center, Department of Neurology, Northwestern University Feinberg School of Medicine, Chicago, IL, USA, while the SK-N-SH cell-line was provided by Justyna Leja, Department of Immunology, Genetics and Pathology, Uppsala University, Uppsala, Sweden. The veracity of the cell-lines was checked in experiments, where it could be confirmed that HOG and MO3.13 expressed 2′,3′-cyclic-nucleotide 3′-phosphodiesterase (CNPase) and SK-N-SH expressed neural cell adhesion molecule 2 (NCAM2).

### Cell culture

The HOG and MO3.13 cells were maintained in Dulbecco’s modified eagle medium (Thermo Fisher Scientific; cat#10313021) supplemented with 10% fetal bovine serum (Thermo Fisher Scientific; cat#10500064), 2 mM l-glutamine (Merck; cat # G7513-100ML) and 1× Penicillin-Streptomycin Solution (Thermo Fisher Scientific; cat# 15140122). The SK-N-SH cells were thawed and expanded in RPMI-1640 media (Thermo Fisher Scientific; cat # 21870076) supplemented with 10% fetal bovine serum, 2 mM l-glutamine and 1× penicillin-streptomycin solution. All cell cultures were grown as monolayers in either T75 or T150 tissue culture flasks (Corning^®^; cat# 15350591 and cat# 10585901, respectively) and maintained in a humidified incubator with 5% CO_2_ at 37°C. The media was changed twice a week.

The cell-lines were passaged when they had reached 80–90% confluency using either trypsin (Thermo Fisher Scientific; cat# 25300054) for SK-N-SH and MO3.13 cells and accutase (Thermo Fisher Scientific; cat# A1110501) for the HOG cells. HOG and MO3.13 cells were split in a 1:6–1:9 ratio whereas the SK-N-SH cells were split in a 1:3–1:6 ratio.

### Immunocytochemistry

For immunocytochemistry (ICC), cells were cultured on eight well μ-slides (Ibidi; cat# 80826) and fixed in 4% paraformaldehyde diluted in phosphate buffer saline (PBS) for 10 min at room temperature. Thereafter, cells were incubated with block buffer [either 5% goat serum (Thermo Fisher Scientific; cat# 31873) for CNPase and NCAM2 antibodies or 10% goat serum for CSF samples; both diluted in 1% bovine serum albumin (BSA)-PBS]. Primary antibodies, NCAM2 (1:250) and CNPase (1:150) were diluted in block buffer. Equal volumes of CSF from healthy controls and MS patients were added to the desired wells and all incubated at 4°C overnight. The next day cells were washed with PBS and incubated with secondary alexa488 conjugates (goat anti-rabbit or goat anti-human) at room temperature for 1 h followed by washing in PBS. The cells were mounted with mounting media containing 4',6-diamidino-2-phenylindole (DAPI) (Ibidi; cat# 50011) and imaged using Zeiss LSM700 inverted confocal microscope with 40× objective using ZEN2000 software (Zeiss). Confocal images were analysed using ImageJ (NIH).

### Cell-based ELISA

The cells were dissociated from the T150 flask using trypsin or accutase, followed by washing in ice cold PBS [ (Thermo Fisher Scientific; cat#14190144)]. The cells were resuspended in PBS to a concentration of 5 × 10^5^ cells/mL and 5 × 10^4^ cells were added to each well in a cone-bottom 96-well plate (Thermo Fisher Scientific; cat#10430153). The plates were centrifuged at 470 *× g* for 3 minutes at 4°C and the supernatant was discarded. The cells were then fixated for 10 minutes using ice cold 1% Paraformaldehyde [Pierce™ 16% Formaldehyde (w/v), Methanol-free, Thermo Fisher Scientific; cat# 28906] diluted in PBS. Thereafter, the cells were washed twice in PBS followed by blocking for 45 minutes at room temperature with 5% bovine serum albumin (BSA, Merck; cat# A7906–100G) diluted in PBS-Tween [(PBS-T (0.1%)]. The plates were placed in a shaker.

Meanwhile, plasma and CSF samples were thawed on ice. Plasma samples and experimental controls as well as some of the CSF samples were reconstituted with 1% BSA diluted in PBS-T (0.1%) (1% BSA-PBS-T; wash buffer). Plasma samples were diluted to a 1:20, ratio; while some of the CSF samples were diluted to a ratio of 1:5 (only where indicated). Other CSF samples were not diluted.

Equal amounts of diluted plasma (or CSF) samples, blanks and controls were added to appropriate wells on a V-bottom 96-well plate (Thermo Fisher Scientific; cat# 249662). For SK-N-SH, neural cell adhesion molecule 2 antibody [NCAM2 (Thermo Fisher Scientific; cat# PA5-112624)] and for HOG and MO3.13 cell-lines, 2′,3′-cyclic-nucleotide 3′-phosphodiesterase antibody [CNPase (Thermo Fisher Scientific; cat# PA5-11089)] was used as a positive control at a final concentration of 1 µg/mL. The plates were incubated on a shaker for an hour at room temperature.

Biotinylated anti-human IgG and IgM antibodies, diluted in wash buffer at a concentration of 1 µg/mL, were incubated separately for IgG and IgM detection. A biotinylated anti-rabbit antibody was used for the detection of the experimental control antibody. Thereafter, the plates were washed and incubated with Avidin conjugated with horse radish peroxide (Avidin-HRP; Agilent, cat# P0364) at 4°C for 45 minutes in the dark on a shaker and washed thrice in 1% BSA diluted in PBS.

Avidin-HRP activity was detected by 3,3′,5,5′-tetramethylbenzidine (TMB) substrate kit (Thermo Fisher Scientific; cat# 34021) containing TMB and peroxide solution mixed in a 1:1 ratio. Equal amounts of TMB substrate were added to each well and incubated at room temperature for 10–15 minutes. Once a strong blue colour had formed, an equal amount of stop solution (Thermo Fisher Scientific; cat# N600) was added. Thereafter, the plates were centrifuged at 470 *× g* for 3 minutes and equal amounts of supernatant were transferred to a flat bottom 96-well plate (Merck; cat# CLS3590). The plates were read at 450 nm using a microplate reader (Biochrom EZ Read 400; Cambridge, UK).

### Statistical analysis

All samples were run in triplicate and the mean values of the triplicates were calculated and used for further analysis. Normality was assessed by Shapiro–Wilk’s test. The statistical significance of the difference between the two groups was calculated using the Mann–Whitney test (A and B panels of Figs 3-8). Kruskal–Wallis test was used to establish statistical significance comparing multiple groups followed by Dunn’s multiple comparison tests for post-hoc analysis (C and D panels of Figs 3–8 and Supplementary Fig. 3). One-way analysis of variance followed by Geisser–Greenhouse correction was used to establish statistical significance comparing multiple groups (Supplementary Fig. 4). Statistical significances were denoted *P < 0.05, **P < 0.01, ***P < 0.001 and ****P < 0.0001. Statistical analyses were performed either using SPSS (IBM SPSS Statistics for Windows, Version 22.0. Armonk, NY: IBM Corp) or GraphPad Prism 7 (GraphPad Software, Inc.).

## Results

### Dynamic range and serial dilutions of control antibodies, sera and CSF

The working concentrations of control antibodies were determined using serial dilutions of CNPase and NCAM2 (10 µg/mL, 1 µg/mL, 0.1 µg/mL, 0.01 µg/mL) binding to HOG and SK-N-SH cells, respectively. The control antibodies concentration reached a plateau at lower concentrations than 0.1 µg/mL. The dilution curves for CNPase and NCAM2 antibodies are shown in Supplementary Figs 1 and 2, respectively.

The dynamic range of the cell-based ELISA assay for IgG and IgM antibodies was determined using serial dilutions of control/MS sera (1:20, 1:200, 1:2000 and 1:20 000) and CSF samples (undiluted CSF, 1:5 and 1:50). The signals gradually diminished at lower concentrations resulting in lower signal to noise ratio. A serial dilution of 1:20 for sera samples and undiluted CSF (data not shown) performed best in the experimental set-up. Therefore, the results mentioned in this study were based on 1:20 dilution for sera and undiluted for CSF analysis. A 1:5 CSF dilution was used for SK-N-SH cells due to sample limitation. The comparison between sera IgG antibodies binding to HOG cell-lines at 1:20, 1:200, 1:2000 and 1:20 000 are shown in Supplementary Figs 3 and 4. In addition to determining dilutions for control antibodies and sera/CSF samples, we also assessed the endogenous peroxidase activity; where, we could not detect any significant peroxidase activity stemming from the cell-lines or the matrix alone as shown in the Supplementary material section ‘assessment of endogenous peroxidase activity.’

### Binding of control antibodies and IgG

**Figure 1:**
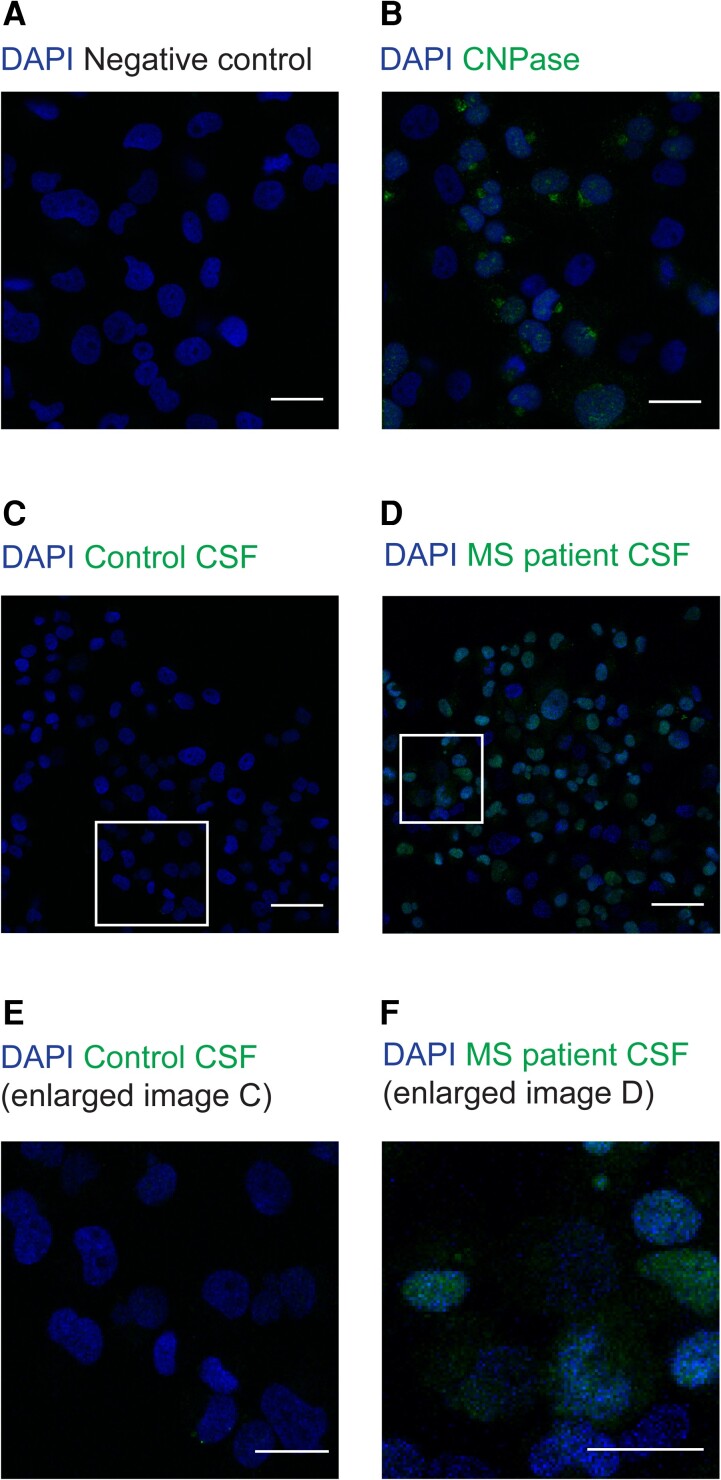
**Immunostainings of HOG cells.** Representative images from ICC staining showing the oligodendroglial cell-line, HOG cells. **(A)** shows negative control for HOG cells. **(B)** shows staining for CNPase, a marker for oligodendrocytes. HOG cells were either incubated with CSF from healthy controls or MS patients and the IgG binding to HOG cells was assessed using anti-human IgG antibody. **(C)** A very weak IgG staining from control CSF was observed, suggesting very low IgG in CSF from healthy individuals. **(D)** An intense IgG staining with CSF from MS patients was observed, suggesting markedly increased IgG as compared to control CSF. **(E and F)** display magnified regions from (**C and D**), respectively. The nuclei was stained with DAPI. Scale bar (A and B) = 25 μm, (C and D) = 50 μm and (E and F) = 25 μm.

First, ICC was performed to assess the expression of CNPase on the HOG cell-line. Immunostainings confirmed the expression of CNPase in HOG cells (Fig. 1A and B). Then we assessed CSF from patients and controls. A very weak staining of IgG from healthy controls’ CSF to HOG cells (Fig. 1C, and an enlarged region of interest in Fig. 1E) was seen, while IgG from MS patients’ CSF bound markedly with an intense IgG staining (Fig. 1D, and enlarged region of interest in Fig. 1F). The mean fluorescent intensities (MFI) for the expression of negative control and CNPase from HOG cell-line (the quantitative analysis for Fig. 1A and B) is shown in Supplementary Fig. 5A, while the MFI for antibodies from control and MS patients’ CSF (the quantitative analysis for Fig. 1C and D) binding to HOG cell-line is shown in Supplementary Fig. 5B.

**Figure 2:**
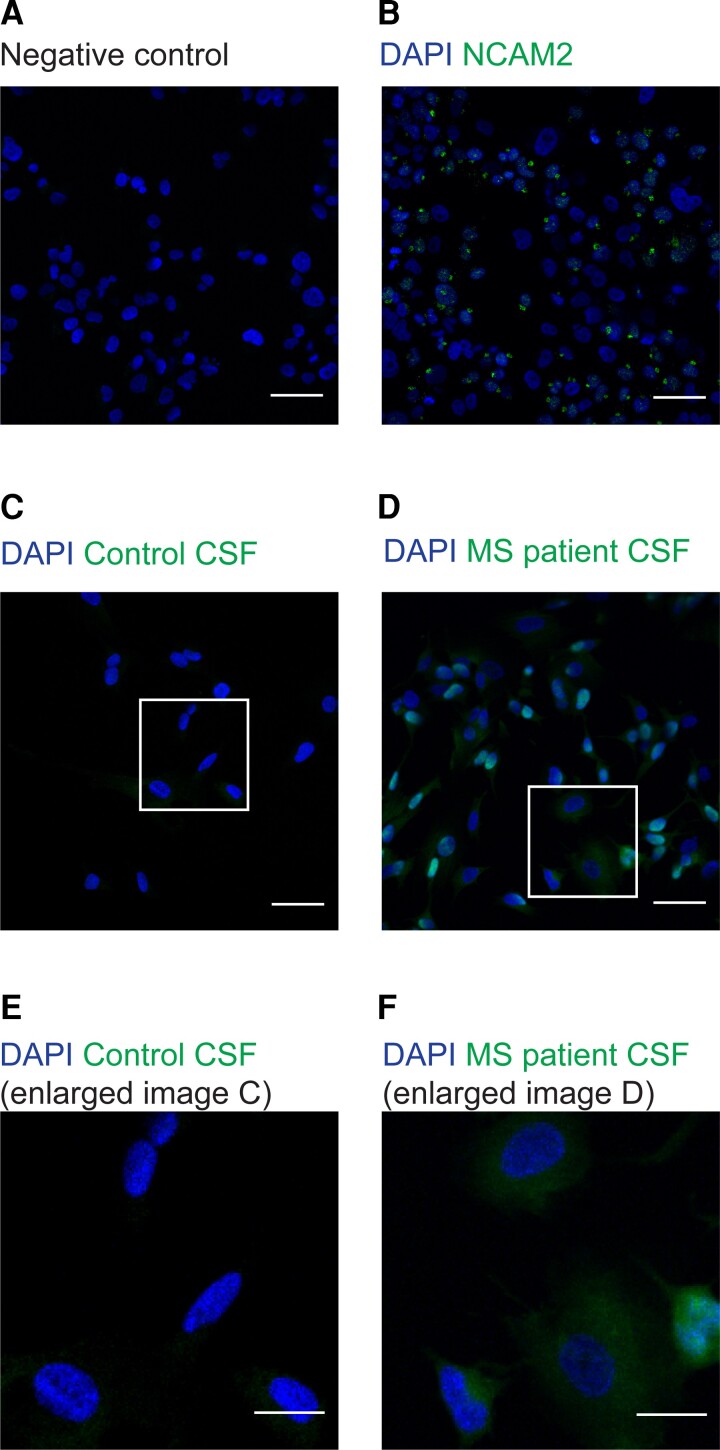
**Immunostainings of SK-N-SH cells.** Representative images from ICC staining with the neuronal cell-line, SK-N-SH cells. **(A)** shows negative control for SK-N-SH cells. **(B)** shows staining for NCAM2, a marker for neuronal cells. SK-N-SH cells were either incubated with CSF from healthy controls or MS patients, the IgG binding to SK-N-SH cells was assessed using anti-human IgG antibody. **(C)** A very weak IgG staining from control CSF was observed, suggesting very low IgG in CSF from healthy individuals. **(D)** An intense IgG staining with CSF from MS patients was observed, suggesting a markedly increase IgG as compared to control CSF. **(E and F)** display magnified regions from **(C and D)**, respectively. The nuclei is stained with DAPI. Scale bar (A–D) = 50 μm and (E and F) = 25 μm.

Similarly, ICC was performed to assess the expression of NCAM2. Immunostaining confirmed the expression of NCAM2 in SK-N-SH cells (Fig. 2A and B). Again, a very weak staining of IgG from healthy controls’ CSF to SK-N-SH cells (Fig. 2C, and enlarged region of interest in Fig. 2E) was seen, while IgG from MS patients’ CSF bound markedly with an intense IgG staining (Fig. 2D, and enlarged region of interest in Fig. 2F). The MFI for the expression of negative control and NCAM2 from SK-N-SH cell-line (the quantitative analysis for Fig. 2A and B) is shown in Supplementary Fig. 6A, while the MFI for antibodies from control and MS patients’ CSF (the quantitative analysis for Fig. 2C and D) binding to SK-N-SH cell-line is shown in Supplementary Fig. 6B.

### Antibodies from MS sera and CSF binds highly to oligodendroglial cell-lines in a cell-based ELISA

The HOG and MO3.13 cell-lines express CNPase, an oligodendrocytic marker, which was used as an experimental control to minimize the effect of plate-to-plate variation. All data used for antibody binding to HOG and MO3.13 cell-lines were normalized to CNPase expression.

#### IgG binding to HOG cells

**Figure 3:**
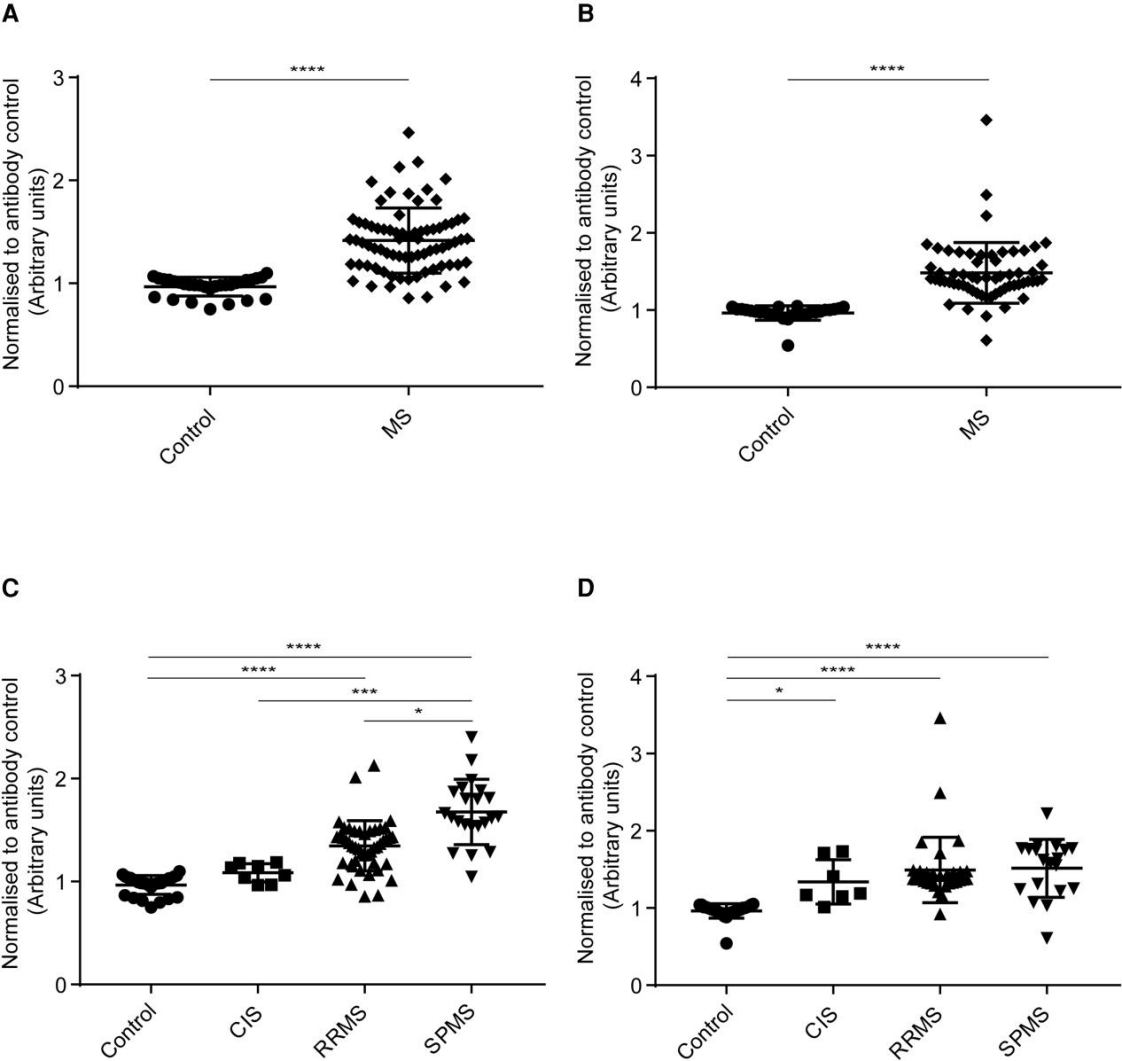
**Human IgG binding to HOG cells.** Cell-based ELISA measurements of human IgG from sera **(A and C)** and CSF **(B and D)** of healthy controls and MS patients (CIS, RRMS and SPMS) binding to oligodendrocytic cell-line (HOG cells) are shown. **(A)** IgG from the serum of MS patients (all three MS subsets combined) bound significantly more to the HOG cell-line than IgG in serum from controls. **(B)** IgG from CSF of MS patients (all three MS subsets combined) bound significantly more to HOG cell-line than IgG in serum from controls. **(C)** IgG from the serum of SPMS patients bound more to HOG cell-line than the other groups. IgG from serum bound significantly higher between control and RRMS, control and SPMS, CIS and SPMS, and RRMS and SPMS. **(D)** IgG from CSF of RRMS and SPMS patients bound more to HOG cell-line than control and CIS groups. IgG from CSF bound significantly higher between, control and CIS, control and RRMS, and control and SPMS. The data shown were normalized to a positive control antibody, CNPase; data are shown as normalized mean values with error bars as standard deviations. The statistical significance of the difference between the groups was calculated using the Mann–Whitney test **(A and B)** and the Kruskal–Wallis test, followed by Dunn’s multiple comparison tests **(C and D)**. *P < 0.05; ***P < 0001; ****P < 0.0001.

**Figure 4:**
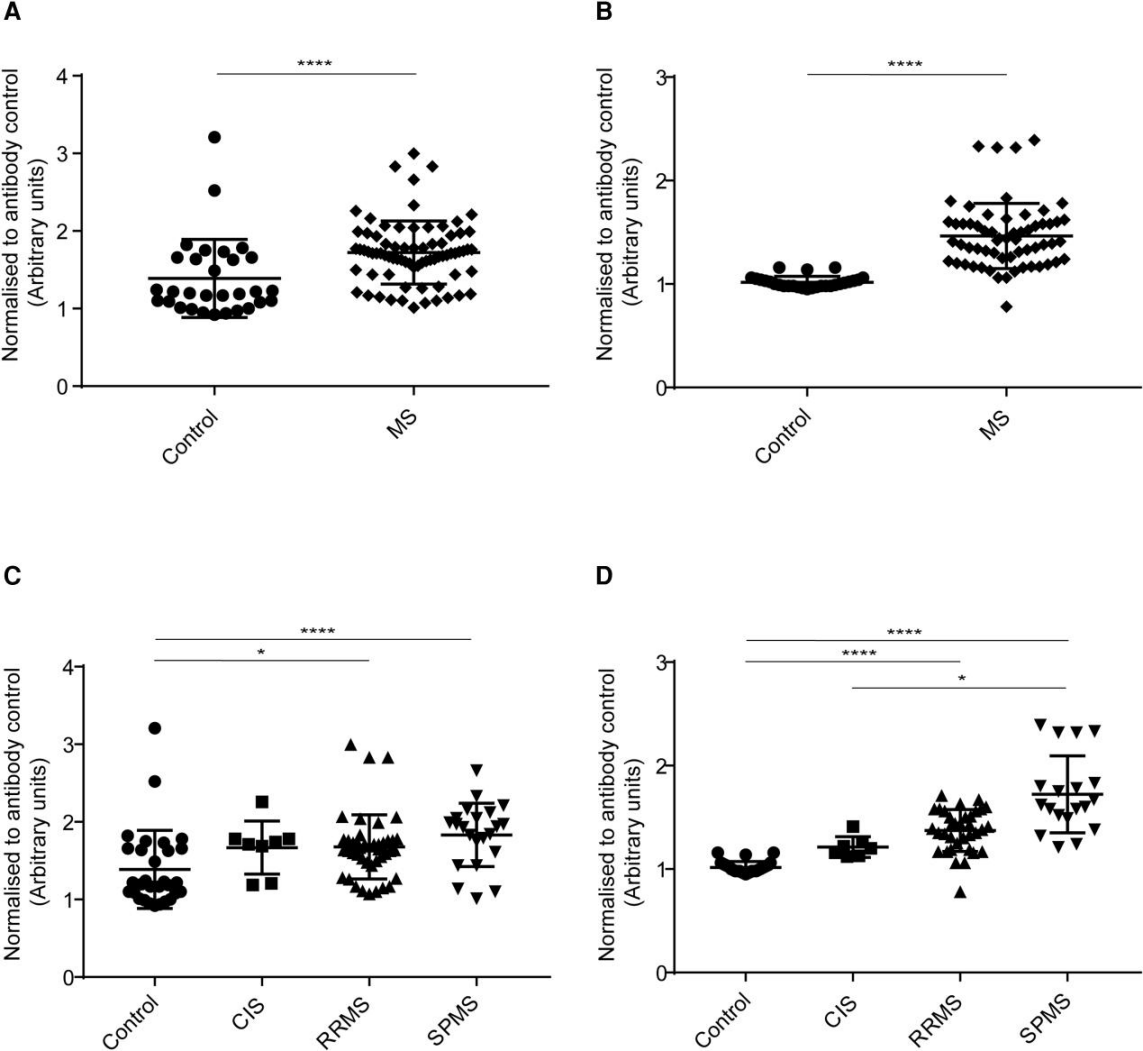
**Human IgM binding to HOG cells.** Cell-based ELISA measurements of human IgM from sera **(A and C)** and CSF **(B and D)** of healthy controls and MS patients (CIS, RRMS and SPMS) binding to oligodendrocytic cell-line (HOG cells) are shown. **(A)** IgM from serum of MS patients (all three MS subsets combined) bound significantly more to HOG cell-line than IgG in serum from controls. **(B)** IgM from CSF of MS patients (all three MS subsets combined) bound significantly more to the HOG cell-line than IgM in serum from controls. **(C)** IgM from serum bound significantly higher between, control and RRMS, and control and SPMS. **(D)** IgM from CSF of SPMS patients bound more to HOG cell-line than the other groups. IgM from CSF bound significantly higher between, control and RRMS, control and SPMS, and CIS and SPMS. The data shown were normalized to a positive control antibody, CNPase; data are shown as normalized mean values with error bars as standard deviations. The statistical significance of the difference between the groups was calculated using the Mann–Whitney test **(A and B)** and the Kruskal–Wallis test, followed by Dunn’s multiple comparison tests **(C and D)**. *P < 0.05; ****P < 0.0001.

IgG antibodies from MS (all MS subgroups combined) patients’ sera (Fig. 3A) and CSF (Fig. 3B) bound significantly higher than healthy controls. The analysis for sera IgG binding to HOG cells among MS subgroups and control showed a statistically significant difference in binding between control and RRMS, control and SPMS, CIS and SPMS, and RRMS and SPMS subgroups (Fig. 3C). The analysis for CSF IgG binding to HOG cells among MS subgroups and control showed a statistically significant difference in binding between control and RRMS, control and CIS, and control and SPMS (Fig. 4D).

#### IgM binding to HOG cells

IgM antibodies from MS (all MS subgroups combined) patients’ sera (Fig. 4A) and CSF (Fig. 4B) bound significantly higher than healthy controls. The analysis for sera IgM binding to HOG cells among MS subgroups and control showed a statistically significant difference in binding between control and RRMS, and control and SPMS (Fig. 4C). The analysis for CSF IgM binding to HOG cells among MS subgroups and control showed a statistically significant difference in binding between control and RRMS, control and SPMS, and CIS and SPMS subgroups (Fig. 4D).

#### IgG binding to MO3.13 cells

**Figure 5:**
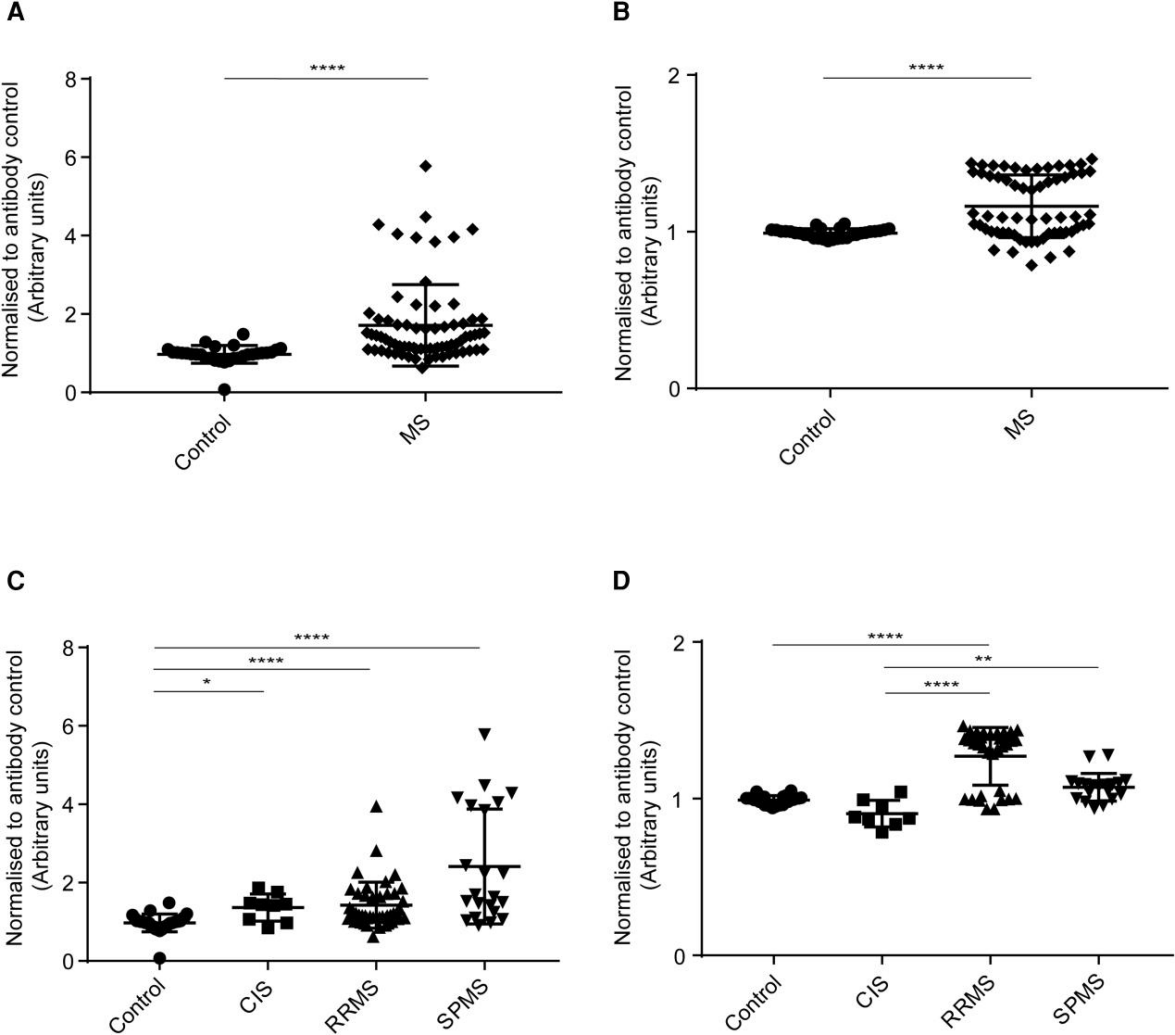
**Human IgG binding to MO3.13 cells.** Cell-based ELISA measurements of human IgG from sera **(A and C)** and CSF **(B and D)** of healthy controls and MS patients (CIS, RRMS and SPMS) binding to oligodendrocytic cell-line (MO3.13 cells) are shown. **(A)** IgG from serum of MS patients (all three MS subsets combined) bound significantly more to MO3.13 cell-line than IgG in serum from controls. **(B)** IgG from CSF of MS patients (all three MS subsets combined) bound significantly more to MO3.13 cell-line than IgG in serum from controls. **(C)** IgG from serum bound significantly higher between, control and CIS, control and RRMS and control and SPMS. **(D)** IgG from serum bound significantly higher between, control and RRMS, CIS and RRMS, and CIS and SPMS. The data shown were normalized to a positive control antibody, CNPase; data are shown as normalized mean values with error bars as standard deviations. The statistical significance of the difference between the groups was calculated using the Mann–Whitney test **(A and B)** and the Kruskal–Wallis test, followed by Dunn’s multiple comparison tests **(C and D)**. *P < 0.05; **P < 0.01; ****P < 0.0001.

IgG antibodies from MS (all MS subgroups combined) patients’ sera (Fig. 5A) and CSF (Fig. 5B) bound significantly higher than healthy controls. The analysis for sera IgG binding to MO3.13 cells among MS subgroups and control showed a statistically significant difference in binding between control and CIS, control and RRMS, and control and SPMS (Fig. 5C). The analysis for CSF IgG binding to MO3.13 cells among MS subgroups and control showed a statistically significant difference in binding between control and RRMS, CIS and RRMS, and CIS and SPMS subgroups (Fig. 5D).

#### IgM binding to MO3.13 cells

**Figure 6:**
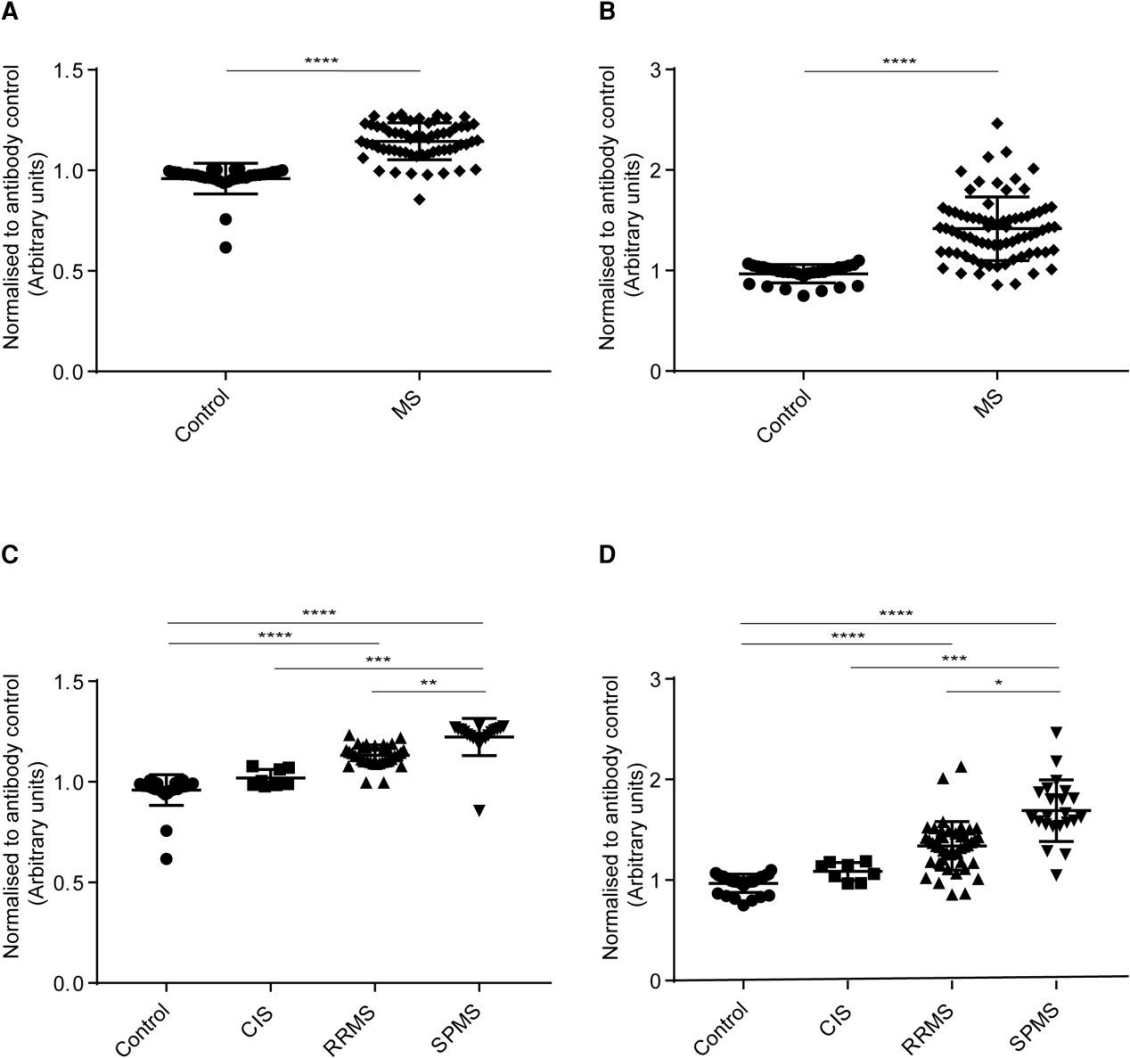
**Human IgM binding to MO3.13 cells.** Cell-based ELISA measurements of human IgM from sera **(A and C)** and CSF **(B and D)** of healthy controls and MS patients (CIS, RRMS and SPMS) binding to oligodendrocytic cell-line (MO3.13 cells) are shown. **(A)** IgM from serum of MS patients (all three MS subsets combined) bound significantly more to MO3.13 cell-line than controls. **(B)** IgM from CSF of MS patients (all three MS subsets combined) bound significantly more to MO3.13 cell-line than controls. **(C)** IgM from serum of SPMS patients bound more to MO3.13 cell-line than the other groups. IgG from serum bound significantly higher between, control and RRMS, control and SPMS, CIS and SPMS, and RRMS and SPMS. **(D)** IgM from CSF of SPMS patients bound more to MO3.13 cell-line than the other groups. IgM from CSF bound significantly higher between, control and RRMS, control and SPMS, CIS and SPMS, and RRMS and SPMS. The data shown were normalized to a positive control antibody, CNPase; data are shown as normalized mean values with error bars as standard deviations. The statistical significance of the difference between the groups was calculated using the Mann–Whitney test **(A and B**) and the Kruskal–Wallis test, followed by Dunn’s multiple comparison tests **(C and D)**. *P < 0.05; **P < 0.01; ***P < 0001; ****P < 0.0001.

IgM antibodies from MS (all MS subgroups combined) patients’ sera (Fig. 6A) and CSF (Fig. 6B) bound significantly higher than healthy controls. The analysis for sera IgM binding to MO3.13 cells among MS subgroups and control showed a statistically significant difference in binding between control and RRMS, control and SPMS. CIS and SPMS, and RRMS and SPMS subgroups (Fig. 6C). The analysis for CSF IgM binding to MO3.13 cells among MS subgroups and control showed a statistically significant difference in binding between control and RRMS, control and SPMS, CIS and SPMS, and RRMS and SPMS subgroups (Fig. 6D).

### Antibodies from MS sera and CSF binds highly to neuronal cell-line in a cell-based ELISA

SK-N-SH cell-line express NCAM2, a neuronal marker, which was used as an experimental control to minimize the effect of plate-to-plate variation. All data used for antibodies binding to the SK-N-SH cell-line were normalized to NCAM2 expression.

#### IgG binding to SK-N-SH cells

**Figure 7:**
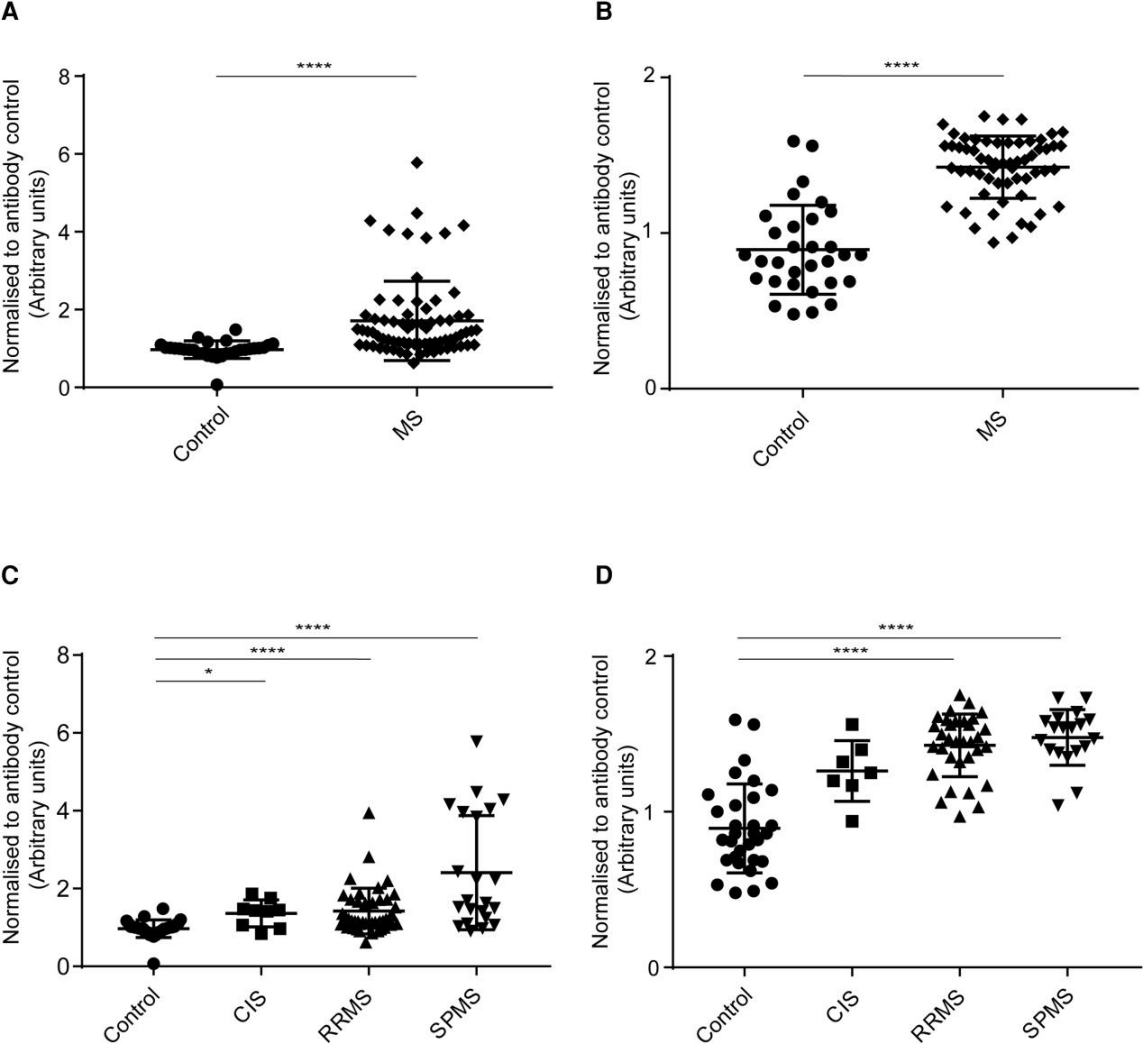
**Human IgG binding to SK-N-SH cells.** Cell-based ELISA measurements of human IgG from sera **(A and C)** and CSF **(B and D)** of healthy controls and MS patients (CIS, RRMS and SPMS) binding to a neuronal cell-line (SK-N-SH cells) are shown. **(A)** IgG from the serum of MS patients (all three MS subsets combined) bound significantly more to SK-N-SH cell-line than controls. **(B)** IgG from CSF of MS patients (all three MS subsets combined) bound significantly more to SK-N-SH cell-line than controls. **(C)** A trend towards the increased binding of IgG from serum of control to CIS to RRMS to SPMS groups was found, where SPMS bound the highest to SK-N-SH cell-line as compared to other groups. IgG from serum bound significantly higher between, control and CIS, control and RRMS, and control and SPMS. **(D)** A trend towards the increased binding of IgG from CSF of control to CIS to RRMS to SPMS groups was found, where SPMS bound the highest to SK-N-SH cell-line as compared to other groups. IgG from CSF bound significantly higher between, control and RRMS, and control and SPMS. The data shown were normalized to a positive control antibody, NCAM2; data are shown as normalized mean values with error bars as standard deviations. The statistical significance of the difference between the groups was calculated using the Mann–Whitney test **(A and B)** and the Kruskal–Wallis test, followed by Dunn’s multiple comparison tests **(C and D)**. *P < 0.05; ****P < 0.0001.

IgG antibodies from MS (all MS subgroups combined) patients’ sera (Fig. 7A) and CSF (Fig. 7B) bound significantly higher than healthy controls. The analysis for sera IgG binding to SK-N-SH cells among MS subgroups and control showed a statistically significant difference in binding between control and CIS, control and RRMS, and control and SPMS (Fig. 7C). The analysis for CSF IgG binding to SK-N-SH cells among MS subgroups and control showed a statistically significant difference in binding between control and RRMS, and control and SPMS (Fig. 7D).

#### IgM binding to SK-N-SH cells

**Figure 8:**
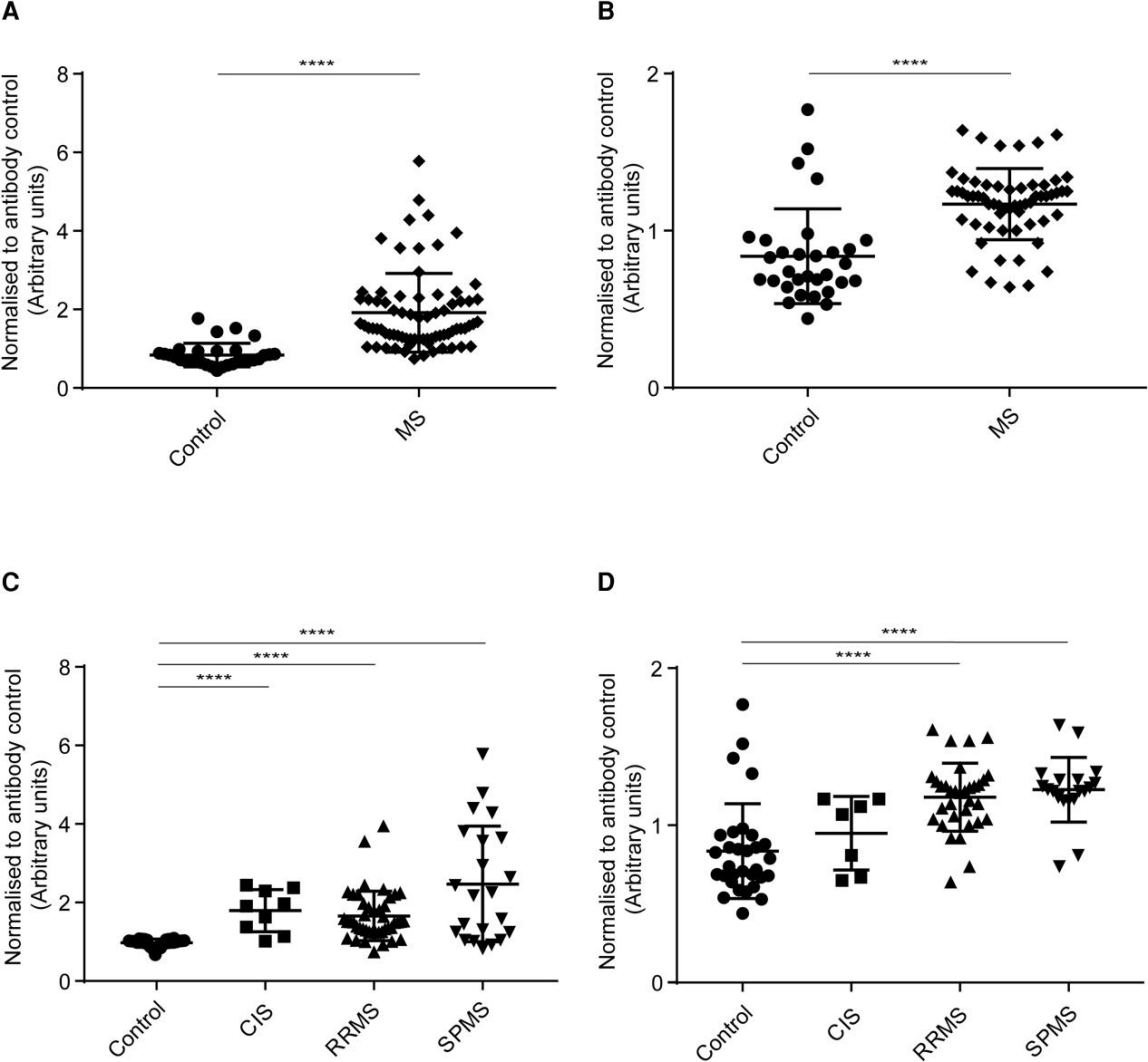
**Human IgM binding to SK-N-SH cells.** Cell-based ELISA measurements of human IgM from sera **(A and C)** and CSF **(B and D)** of healthy controls and MS patients (CIS, RRMS and SPMS) binding to a neuronal cell-line (SK-N-SH cells) are shown. **(A)** IgM from serum of MS patients (all three MS subsets combined) bound significantly more to SK-N-SH cell-line than controls. **(B)** IgM from CSF of MS patients (all three MS subsets combined) bound significantly more to SK-N-SH cell-line than controls. **(C)** A trend towards the increased binding of IgM from serum of control to CIS to RRMS to SPMS groups was found, where SPMS bound the highest to SK-N-SH cell-line as compared to other groups. IgM from CSF bound significantly higher between, control and CIS, control and RRMS, and control and SPMS. **(D)** IgM from CSF bound significantly higher between, control and RRMS, and control and SPMS. The data shown were normalized to a positive control antibody, NCAM2; data are shown as normalized mean values with error bars as standard deviations. The statistical significance of the difference between the groups was calculated using the Mann–Whitney test **(A and B)** and the Kruskal–Wallis test, followed by Dunn’s multiple comparison tests **(C and D)**. ***P < 0001; ****P < 0.0001.

IgM antibodies from MS (all MS subgroups combined) patients’ sera (Fig. 8A) and CSF (Fig. 8B) bound significantly higher than healthy controls. The analysis for sera IgM binding to SK-N-SH cells among MS subgroups and control showed a statistically significant difference in binding between control and CIS, control and RRMS, and control and SPMS (Fig. 8C). The analysis for CSF IgM binding to SK-N-SH cells among MS subgroups and control showed a statistically significant difference in binding between control and RRMS, and control and SPMS (Fig. 8D).

### Specificity and sensitivity

We have performed specificity and sensitivity analysis on our cell-based ELISA data to understand its strength and shortcomings as well as analysis for likelihood ratio (LR) to determine its potential utility in diagnostic tests. The specificity and sensitivity, the LR, an area under the curve, cut-off values and the significance of immunoglobulins from MS sera/CSF (compared to control sera/CSF) binding to each cell-line are summarized in Table 1.

**Table 1:** The sensitivity, specificity and LR for MS sera/CSF-ig binding to each cell-line compared to controls is shown

Cell-line Ig	Area under the curve	*P*-value	Cut-off	Sensitivity (%)	Specificity (%)	LR
HOG-sera IgG	0.93	<0.0001	>1.084	85.3	96.6	25.6
HOG-CSF IgG	0.96	<0.0001	>1.045	93.4	96.4	26.2
HOG-sera IgM	0.75	<0.0001	>1.825	30.7	93.3	4.6
HOG-CSF IgM	0.97	<0.0001	>1.150	91.8	92.8	12.8
MO3.13-sera IgG	0.85	<0.0001	>1.298	52.1	96.7	15.6
MO3.13-CSF IgG	0.75	<0.0001	>1.047	61.3	96.7	18.4
MO3.13-sera IgM	0.94	<0.0001	>1.084	85.3	96.7	25.6
MO3.13-CSF IgM	0.95	<0.0001	>1.007	87.1	96.9	27.9
SK-N-SH-sera IgG	0.92	<0.0001	>1.340	74.6	93.5	11.6
SK-N-SH-CSF IgG	0.85	<0.0001	>1.298	52.7	96.7	15.8
SK-N-SH-sera IgM	0.82	<0.0001	>0.990	84.8	87.1	6.6
SK-N-SH-CSF IgM	0.93	<0.0001	>0.525	50.7	96.8	15.7

## Discussion

In this study, we provide data in support of those antibodies from RRMS and SPMS patients’ sera and CSF samples bind to oligodendroglial (HOG and MO3.13) and neuroblastoma (SK-N-SH) cell-lines, compared to controls. Our results suggest that the oligodendroglial and neuroblastoma cell-lines express antigens on their cell surface that bind antibodies from MS patients’ sera and CSF. In general, the differences were larger with CSF samples and the binding was more pronounced with serum and CSF from patients with SPMS.

The pathogenesis of MS is complex and only partially understood. B cells as well as T cells have been implicated in MS pathogenesis,^16^ B cells and plasma cells are detected in active and chronic MS lesions.^17^ B cells are also reported to play a role through antibody-independent mechanisms that include antigen presentation, co-stimulation, and cytokine and chemokine production.^18,19^ In one study of MS patients undergoing treatment with the B-cell depleting agent rituximab, no changes in CSF IgG levels or oligoclonal bands (OCBs) could be demonstrated. This suggests that OCBs are not dependent on B cells and that the effectiveness of rituximab (and other disease modifying drugs) may be independent of CSF IgG levels.^19^

The demonstration of antibodies against either full-length KIR4.1 [(inwardly rectifying potassium (Kir) channel subunit Kir4.1] protein or peptide was considered a breakthrough in the research on MS since antibodies towards KIR4.1 were detected by ELISA in as many as 47% of serum samples obtained from adults with MS or CIS.^20^ However, several studies using identical methods as well as diverse techniques failed to replicate this association.^21,22^ Such studies have methodologic and technical challenges; particularly, those associated with post-translational modifications and higher-order structure formation of the KIR4.1 protein.^21^ A proof-of-concept study was recently designed to use human protein microarrays to identify blood-based antibody capable of diagnosing MS. In this study antibody biomarkers effectively differentiated MS subjects from normal and breast cancer controls, but not from subjects with Parkinson’s disease.^23^ However, protein microarrays involve antigen binding to plate surfaces in two-dimension (2D) conformation, which is not the case with antigens expressed on cell surfaces, which often require native structure and 3D conformation to bind to their molecular target. Therefore, cell-based assays with preserved 3D binding sites may give crucial insights into the optimal interactions between antigens and antibodies.

Our study is a continuation of a previous study made by Lily *et al.*^14^ They used flow cytometry to investigate IgG binding to oligodendrocyte precursor, astrocyte, neuronal, striated muscle and endothelial cell-lines and found that antibodies in sera from MS patients bound to some cell-lines, including HOG, MO3.13 and SK-N-SH. When they differentiated the cell-lines to mature cells, there were no significant differences in the binding of antibodies from control and MS sera to these cell-lines. Therefore, in our experimental set-up, we did not use differentiated cells. Furthermore, they thoroughly characterized the cell-lines for the protein expression of relevant cell markers for neuronal, oligodendrocytic, astrocytic and other cells.^14^ We used the same cell-lines in our study, namely, HOG, SK-N-SH and MO3.13 cells and used an antibody to monitor the expression of oligodendrocytic (CNPase) and neuronal (NCAM2) proteins to verify their origin. The differences between control and MS antibodies were only observed by Lily *et al*. when non-permeablized cells (intact cells) were used, suggesting that the antigens were expressed on the cell-surface.^14^

In our study, patients were separated by disease course and included CIS patient samples, which was not a part of the previous study by Lily *et al*.^14^ These patients had a level of antibody binding that was almost indistinguishable from the healthy controls, suggesting that antibody formation is a feature of later stages of MS. Significant differences were found between CIS and RRMS, and CIS and SPMS patients. In general, the trend was that SPMS patients had higher binding than RRMS patients, which could be due to affinity maturation or epitope spreading.

In general, the difference between MS patients and healthy control was a little higher when CSF was investigated. Nevertheless, a strong signal was also obtained with serum, suggesting that it is a likely systemic exposure of circulating antibodies produced by plasma cells residing in the bone marrow, and thus not only a local production from disease induced localized tertiary lymphoid tissues. Amongst the cell-lines and antibodies investigated, measuring IgG on the HOG cell-line incubated with CSF discriminated best between MS and healthy controls with an area under the curve of 0.96, sensitivity of 93%, specificity of 96% and LR of 26. Although these numbers are certainly impressive, the low signal in patients with CIS probably means that this test is of lesser importance for MS diagnostics than demonstration of OCBs.

Analysis of IgG OCBs is widely used in health care today for MS diagnosis; a meta-analysis found that OCB has a specificity of 94% for MS. However, when considering patients with MS or other neuroinflammatory conditions, the specificity fell to 61%, suggesting that many disease processes may lead to the formation of OCBs.^24^ Several efforts have been made to identify the target(s) of these IgG. One of the most convincing studies identified ubiquitous intracellular proteins, including heat shock proteins, not specific to brain tissue, indicating that in MS part of OCBs do not directly mediate tissue injury, but rather a secondary immune response.^25^

MS patients may also have OCBs of other antibody types than IgG. In one study, about 95% of the MS patients displayed IgG OCBs, around 40% also showed intrathecal IgM production, while CSF IgA synthesis was only observed in 13% of cases. Therefore, IgG (predominantly IgG1), and IgM are considered the major contributors to the OCB formation in the CSF.^26,27^ Elevated intrathecal IgM synthesis has been associated with worse disease evolution.^27^ The detection of IgM OCBs in CIS patients was associated with higher risks of conversion to clinically definite MS and in RRMS patients predicted a higher probability of converting to SPMS.^28^ The presence of IgM OCBs has also been associated with frequent relapses, high expanded disability status scale scores, and a higher probability to convert to SPMS.^29^ It has been reported that higher levels of IgM, but not IgG and IgA, were present already at diagnosis in the CSF of MS patients when compared with patients with other neurological diseases. This result corroborates the hypothesis that IgM production occurs from the early stages of MS.^27^

Somewhat surprisingly, the levels of IgG and IgM were fairly similar. IgM is an efficient complement fixer and activates the classical complement pathway more potently than IgG; therefore, it can elicit a relatively strong immune response.^30^ In our setting, the IgM levels did not indicate a transient expression, but should rather be viewed as a feature of chronic inflammation. A majority of MS patients have oligoclonal IgG bands, but these are not prognostic, whilst a minority of MS patients have IgM bands and these have been associated with worse prognosis.^31^ Rheumatoid factor (RF) is IgM and has been implicated in inflammatory autoimmune diseases, such as rheumatoid arthritis. A study showed significantly elevated RF levels in the serum of MS patients and in some MS patients’ CSF indicating blood-brain barrier damage or normal diffusion of RF into CSF. This suggests that RF production may be related to underlying pathological mechanisms in at least some MS patients and show pathogen-specific IgM antibodies.^32^ Also, intrathecal IgM seems to respond more to treatment than IgG, suggesting a different origin of the antibody.^33^

The oligodendroglial and neuronal cells are important to understand the pathophysiology and disease mechanisms in MS since myelination by oligodendrocytes on neurons is one of the major events affected in MS.^34^ HOG and MO3.13 cell-lines have been employed as models to understand the cellular and molecular interactions implicated in a number of oligodendrocyte linked diseases, including MS, Krabbe’s disease and schizophrenia.^35^ However, there are conflicting reports about their maturation and myelinating oligodendrocytic phenotype. A recent study focused on using several protocols to differentiate these cell-lines to mature oligodendrocytes and myelinating phenotype was unsuccessful.^35^ Therefore, antibodies binding these cell-lines in our study should be considered as oligodendrocyte precursor cells (OPCs) and not immature/mature myelinating oligodendrocytes. Since these cells are more similar to OPCs than oligodendrocytes; antibodies directed toward OPCs could inhibit remyelination.^35^ The SK-N-SH cell-line used in this study should be considered as immature neuronal cells, since these cells express immature neuronal markers, and lack mature neuronal markers and do not have the potential to release neurotransmitters nor form neuronal network.^36^

Our cell-based ELISA assay had several advantages. Our assay could reliably differentiate between controls and MS sera and CSF binding capabilities to cell-lines, which is key to assay development. It is practically relevant to use ELISA assay compared to flow cytometry for several reasons including specificity and sensitivity, running costs, performing several tests in one run using 96- or 384-well format and better separation between groups. The cancerous cell-lines are easy to maintain and expand as compared to stem cell-derived cell-lines, both in terms of costs and time; however, stem cell-derived cell-lines have their own merits.^37,38^

Nonetheless, this study also had some limitations. The cancerous cell-lines have inherent tumorigenic properties and express tumorigenic epitopes that may not be present under physiological conditions and fixation prior addition of antibodies may alter epitopes and thereby impact antibody binding. Further, our cellular model probably represents precursor/immature cells and conclusions regarding antibody binding to mature cells should not be made. Our cell-based ELISA assay could differentiate between controls and MS patients, sera and CSF antibodies binding to cell-lines. However, the assay delivered only relative differences between the groups and cannot be used for quantitative analysis.

In summary, our approach could demonstrate a high degree of IgG and IgM binding on cell-lines of oligodendroglial and neuronal origin in patients with established MS. The molecular targets for these antibodies remain unknown and further studies should be made to address this issue.

## Supplementary Material

fcad164_Supplementary_Data

## Data Availability

The data analysed during this study are available from the corresponding author, upon reasonable request.

## Abbreviations List

BSA =: bovine serum albumin
CIS =: clinically isolated syndrome
CNPase =: 2′,3′-cyclic-nucleotide 3′-phosphodiesterase
HOG cell-line =: human oligodendroglioma cell-line
HRP =: horseradish peroxidase
ICC =: immunocytochemistry
Ig =: immunoglobulin
KIR4.1 =: inwardly rectifying potassium (Kir) channel subunit Kir4.1
LR =: likelihood ratio
MFI =: mean fluorescent intensity
MOGAD =: myelin oligodendrocyte glycoprotein antibody-associated disease
MS =: multiple sclerosis
NCAM2 =: neural cell adhesion molecule 2
NMO =: neuromyelitis optica
OCBs =: oligoclonal bands
OPCs =: Oligodendrocyte precursor cells
PPMS =: primary progressive multiple sclerosis
RF =: Rheumatoid factor
RRMS =: relapsing-remitting multiple sclerosis
SPMS =: secondary progressive multiple sclerosis
TMB =: 3,3′,5,5′-tetramethylbenzidine
