# Supplementary material for: Antibodies from serum and CSF of multiple sclerosis patients bind to oligodendroglial and neuronal cell-lines
Source: Brain Commun. 2023 May 23;5(3):fcad164. doi: 10.1093/braincomms/fcad164 (PMC10233900; doi:10.1093/braincomms/fcad164)
Supplement: fcad164_Supplementary_Data [file fcad164_supplementary_data.pdf]

Supplementary data

Assessment of endogenous peroxidase activity

To assess possible peroxidase activity from the cell-lines used in our experimental set-up or the matrix (serum and CSF), cell-lines or matrix were incubated with TMB substrate and then the color shift was analyzed. We could not detect any significant peroxidase activity stemming from the cell-lines or the matrix alone.

Supplementary Figure 1. Dose response curve of CNPase antibody.

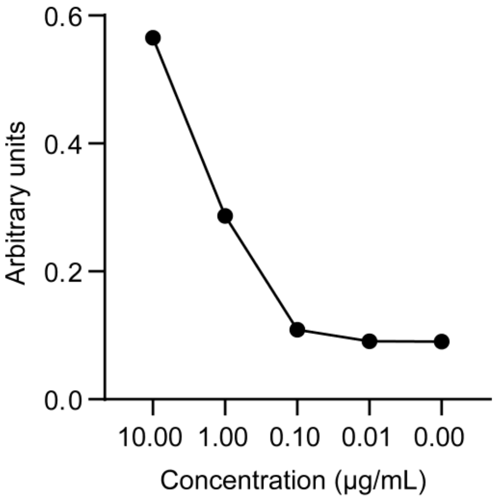

Cell-based ELISA measurements of oligodendrocyte antibody, CNPase at various dilutions. The CNPase antibody binding to HOG cells decreased gradually reaching a plateau at low dilutions of 0.1 and 0.01 µg/mL (0 µg/mL is the blank). A concentration of 1 µg/mL was used for the further experiments, based on these data.

Supplementary Figure 2. Dose response curve of NCAM2 antibody.

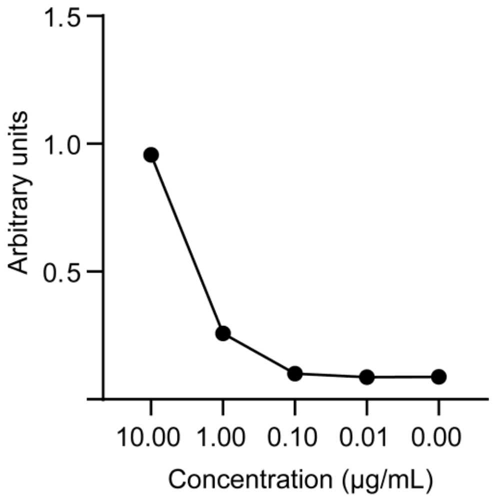

Cell-based ELISA measurements of oligodendrocyte antibody, NCAM2 at various dilutions. The NCAM2 antibody binding decreased gradually to SK-N-SH cells reaching a plateau at low dilutions of 0.1 and 0.01 µg/mL (0 µg/mL is the blank). A concentration of 1 µg/mL was used for the further experiments, based on these data.

Supplementary Figure 3. Effect of serum dilution in the IgG binding to HOG cells.

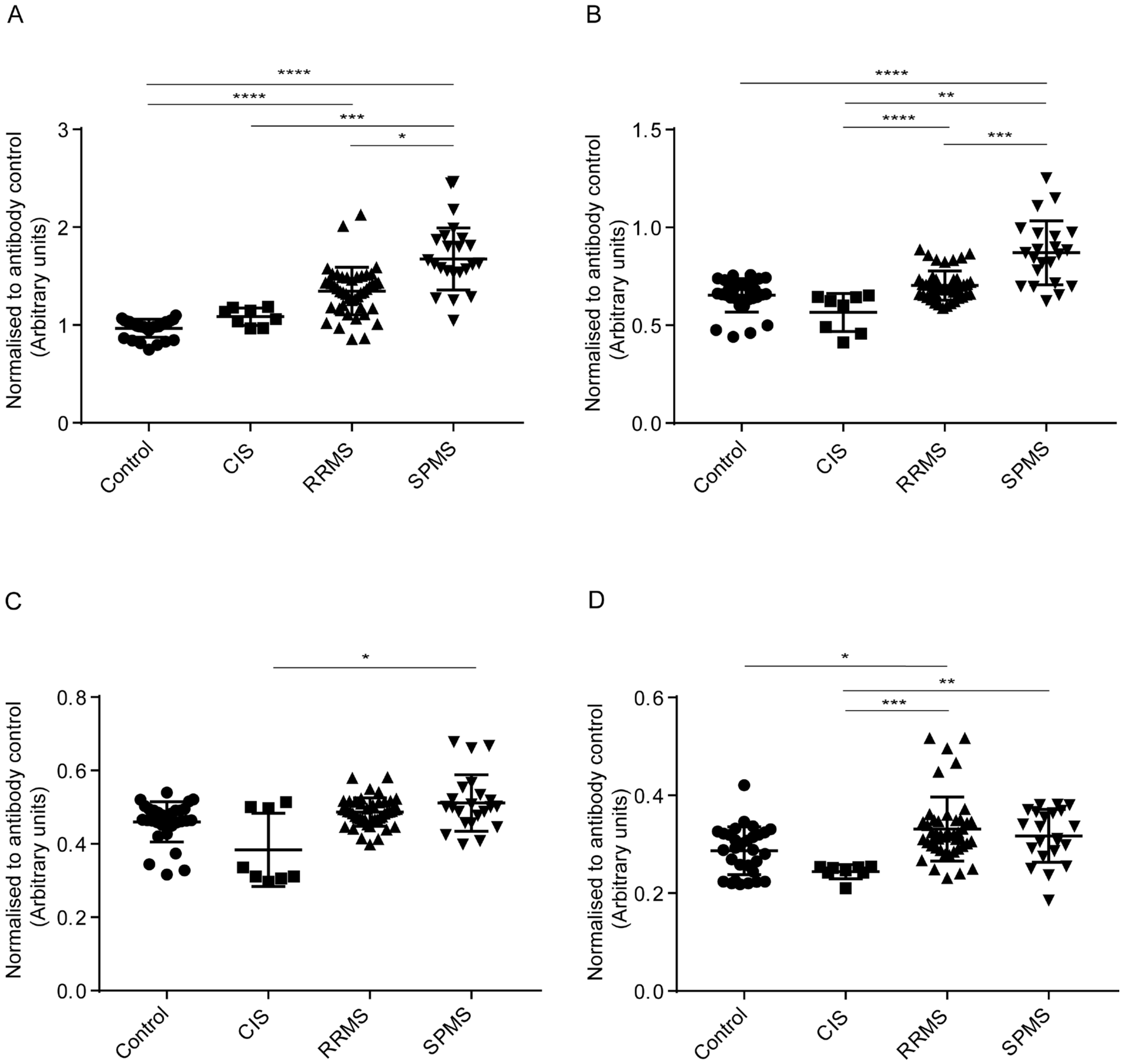

Cell-based ELISA measurements of human sera IgG binding to HOG cells at various sera dilutions of healthy controls and MS patients (CIS, RRMS and SPMS) is shown. IgG binding decreases at higher serum dilution and affects the significant separation between groups. **(A)** IgG from serum of SPMS patients bound more than the other groups. IgG from serum bound significantly higher between control and RRMS, control and SPMS, CIS and SPMS, and RRMS and SPMS at 1:20 serum dilution. **(B)** IgG from serum of SPMS patients bound more than the other groups. IgG from serum bound significantly higher between control and SPMS, CIS and RRMS, CIS and SPMS, and RRMS and SPMS at 1:200 serum dilution. **(C)** IgG from serum bound significantly higher between CIS and SPMS at 1:2000 serum dilution. **(D)** IgG from serum bound significantly higher between control and RRMS, CIS and RRMS, and CIS and SPMS at 1:20000 serum dilution.

The data shown was normalized to a positive control antibody, CNPase; data are shown as normalized mean values with error bars as standard deviations. The statistical significance of the difference between the groups was calculated using the Kruskal-Wallis test, followed by Dunn's multiple comparison test.

* = $p<0.05$; ** = $p<0.01$; *** = $p< 0,001$; **** = $p<0.0001$

Supplementary Figure 4. Dynamic range of serum dilution in the IgG binding to HOG cells.

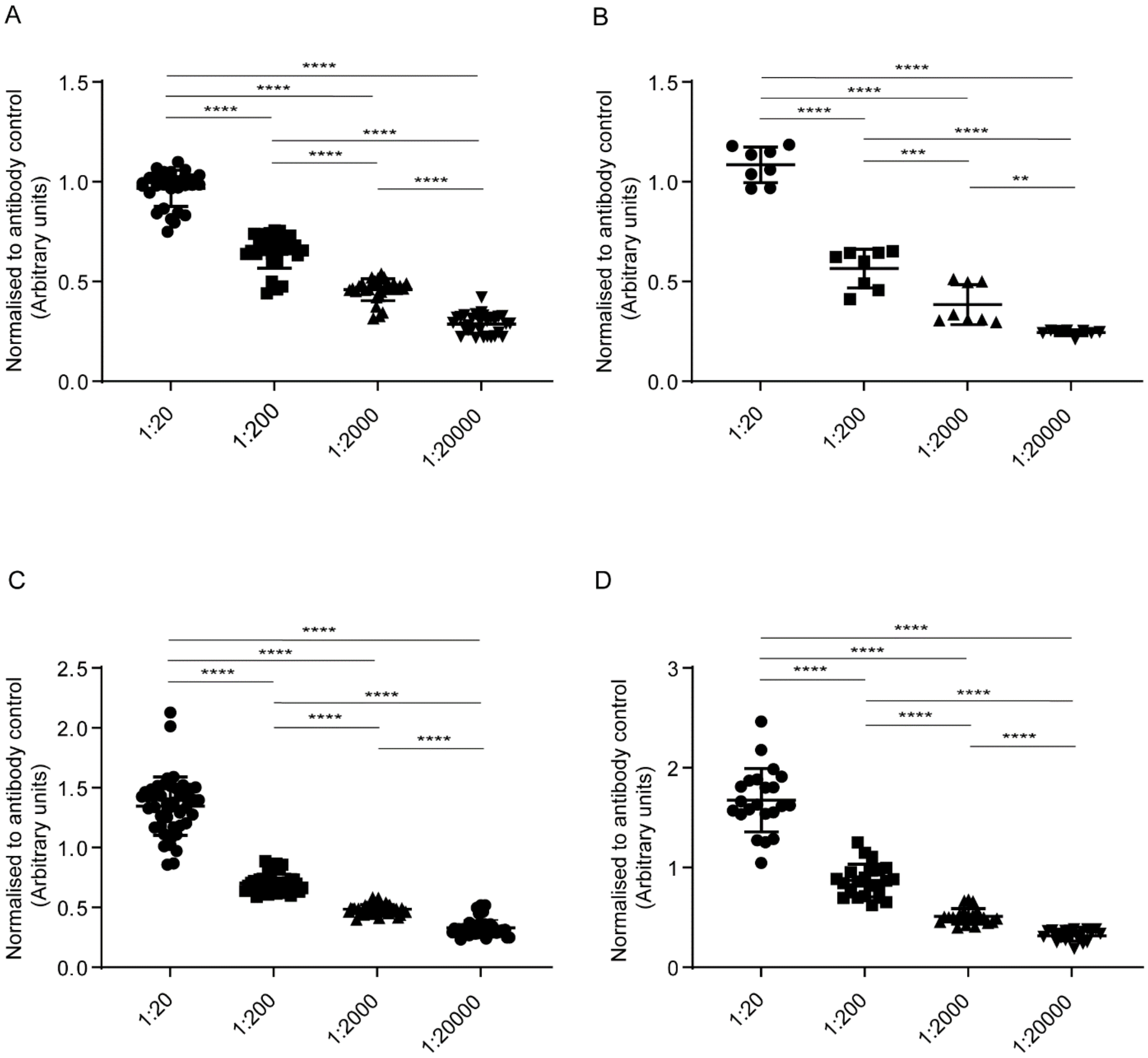

Cell-based ELISA measurements of human sera IgG binding from healthy controls and MS patients (CIS, RRMS and SPMS) to HOG cells at various sera dilutions is shown. **(A)** IgG from serum of healthy controls bound more at 1:20 dilution as compared to serum dilutions at 1:200, 1:2000 and 1:20000. A significant gradual decrease in IgG binding to HOG cells was observed. **(B)** IgG from serum of CIS patients bound more at 1:20 dilution as compared to serum dilutions at 1:200, 1:2000 and 1:20000. A significant gradual decrease in IgG binding to HOG cells was observed. **(C)** IgG from serum of RRMS patients bound more at 1:20 dilution as compared to serum dilutions at 1:200, 1:2000 and 1:20000. A significant gradual decrease in IgG binding to HOG cells was observed. **(D)** IgG from serum of SPMS patients bound more at 1:20 dilution as compared to serum dilutions at 1:200, 1:2000 and 1:20000. A significant gradual decrease in IgG binding to HOG cells was observed.

The data shown was normalized to a positive control antibody, CNPase; data are shown as normalized mean values with error bars as standard deviations. The statistical significance of the difference between the groups was calculated using one-way ANOVA test, followed by Geisser-Greenhouse correction.

** = p<0.01; *** = p< 0,001; **** = p<0.0001

Supplementary Figure 5. Quantitative analysis of CNPase expression and antibodies from CSF binding to HOG cell line.

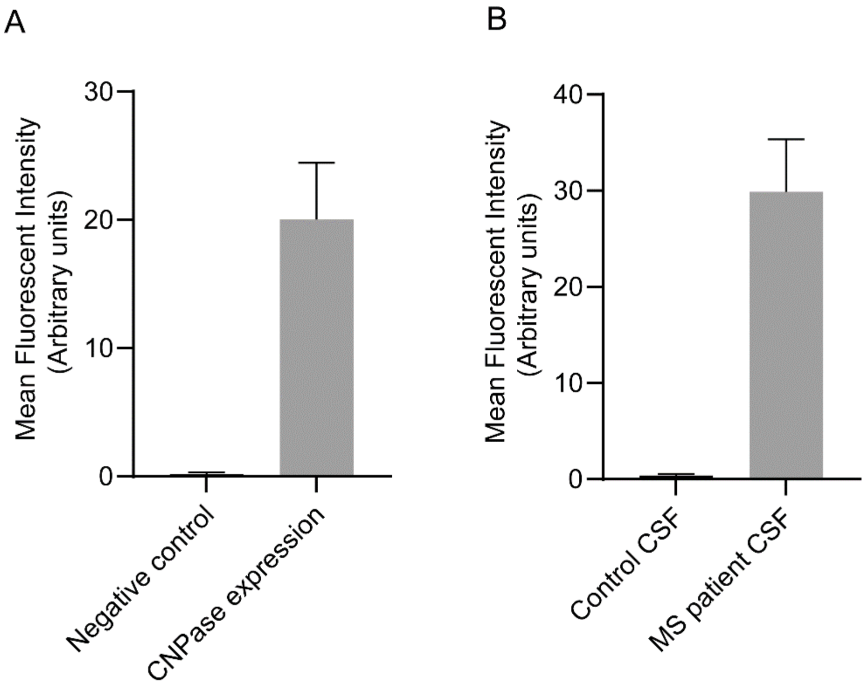

Mean fluorescent intensities for CNPase expression and IgG antibodies from control and MS patients' CSF binding to HOG cell line is shown. **(A)** CNPase, a positive marker for oligodendrocytic cell line is highly expressed in HOG cells, compared to negative control. The bar graph shown in (A) was quantified using images from Figure 1A, B. **(B)** IgG antibodies from MS patients' CSF binds markedly to HOG cells, compared to IgG antibodies from control CSF, The bar graph shown in (B) was quantified using images shown in Figure 1C, D. The error bars represent standard deviation.

Supplementary Figure 6. Quantitative analysis of NCAM2 expression and antibodies from CSF binding to SK-N-SH cell line.

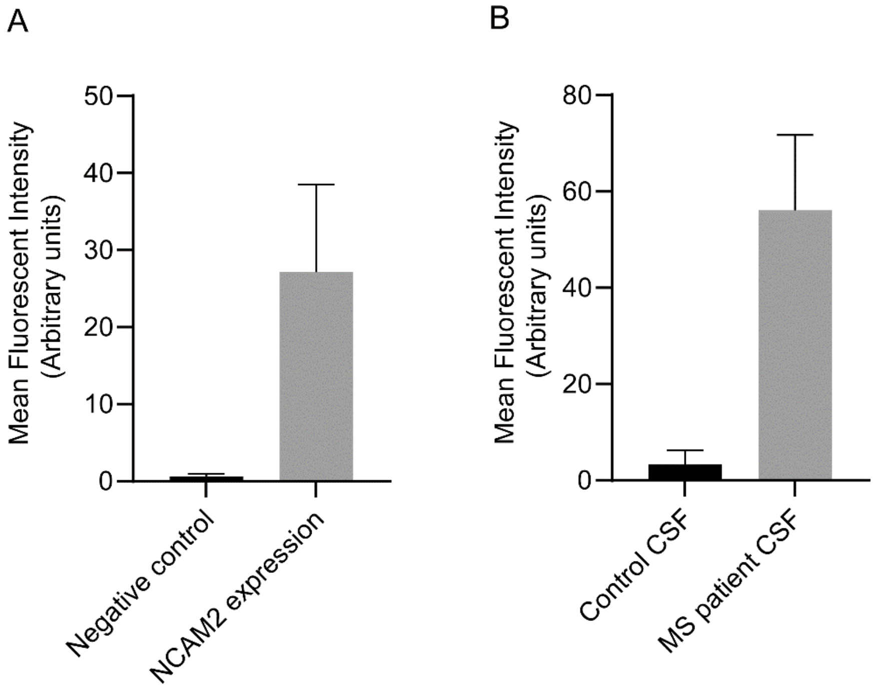

Mean fluorescent intensities for NCAM2 expression and IgG antibodies from control and MS patients' CSF binding to SK-N-SH cell line is shown. **(A)** NCAM2, a positive marker for neuronal cell line is highly expressed in SK-N-SH cells, compared to negative control. The bar graph shown in (A) was quantified using images from Figure 2A, B. **(B)** IgG antibodies from MS patients' CSF binds markedly to SK-N-SH cells, compared to IgG antibodies from control CSF, The bar graph shown in (B) was quantified using images shown in Figure 2C, D. The error bars represent standard deviation.

Supplementary Table 1. Demographic data and clinical characteristics of MS patients and healthy controls (serum samples).

Clinical groups	Controls	CIS	RRMS	SPMS	All MS
Subjects (n)	30	9	45	22	64
Gender % F/M	69/31	67/33	74/26	50/50	66/34
Median age* (range)	35 (19-61)	25 (18-44)	38 (22-70)	62 (35-68)	43 (18-70)
Median disease duration** (range)	Not applicable	3 (0.5-31)	101 (2-346)	256 (2-538)	116 (0.5-538)
Median EDSS (range)	Not applicable	1 (0-3)	2 (0-7.5)	6 (3-7.5)	2.5 (0-7.5)

* years, ** months

Supplementary Table 2. Demographic data and clinical characteristics of MS patients and healthy controls (CSF samples).

* years, ** months

Clinical groups	Controls	CIS	RRMS	SPMS	All MS
Subjects (n)	32	8	35	19	62
Gender % F/M	56/44	62/38	68/32	58/42	65/35
Median age* (range)	48 (19-65)	32 (18-48)	38 (22-70)	62 (35-68)	43.5 (18-70)
Median disease duration** (range)	Not applicable	2.5 (0.5-36)	112 (0.25-346)	280 (113-538)	130 (0.25-538)
Median EDSS (range)	Not applicable	1 (0-3)	2 (0-7.5)	6 (3-7.5)	2 (0-7.5)

Supplementary Table 3. Demographic data, clinical characteristics including, disease duration (months), time from latest relapse (months; days, where mentioned), number of relapses, EDSS score, IgG and IgM index and oligoclonal bands, previous and current treatments of MS patients and healthy controls, are shown for CSF samples, where applicable.

ID	Age	Gender	MS-type	Disease duration	Time from latest relapse	No.of relapses	EDSS	OCB-IgG	IgG-index	OCB-IgM	IgM-index	Treatment	Previous treatment
1	52	M	Ctrl										
2	58	F	Ctrl										
3	26	M	Ctrl										
4	46	F	Ctrl										
5	41	M	Ctrl										
6	51	F	Ctrl										
7	44	M	Ctrl										
8	37	M	Ctrl										
9	26	F	Ctrl										
10	32	F	Ctrl										
11	38	F	Ctrl										
12	19	F	Ctrl										
13	51	F	Ctrl										
14	50	M	Ctrl										
15	48	M	Ctrl										
16	37	F	Ctrl										
17	48	M	Ctrl										
18	61	M	Ctrl										
19	52	F	Ctrl										
20	41	F	Ctrl										
21	56	M	Ctrl										
22	48	F	Ctrl										
23	56	F	Ctrl										
24	35	F	Ctrl										
25	30	F	Ctrl										
26	53	M	Ctrl										
27	42	M	Ctrl										
28	52	M	Ctrl										
29	58	F	Ctrl										
30	65	M	Ctrl										
31	52	F	Ctrl										
32	42	F	Ctrl										
1	20	F	CIS	1	1	1	0	yes	0.93	no	0.092		
2	40	F	CIS	5.5	5.5	1	0	yes	0.87	no			
3	48	F	CIS	36	36	1	1	yes	0.52	no	0.061		
4	25	F	CIS	2	2	1	0	yes (1)	0.48	no	0.77		
5	39	M	CIS	0.5		1	1	yes	0.54	no	0.044		
6	24	F	CIS		1	1	3	yes	0.89	yes (1)	0.183		
7	18	M	CIS		2	1	2		1.63	no	0.313		
8	44	M	CIS	3	3	1	1	yes	0.73	no	0.132		
1	51	F	RRMS	85.5	2 days	7	7.5	yes	1.63			Copaxone	Betaferon
2	39	F	RRMS	196	5	12	2	yes	1.43	no	0.155		Avonex
3	55	F	RRMS	255	27	11	2	yes	0.5	no	0.121		Avonex

ID	Age	Gender	MS-type	Disease duration	Time from latest relapse	No.of relapses	EDSS	OCB -IgG	IgG-index	OCB-IgM	IgM-index	Treatment	Previous treatment
4	44	F	RRMS	99	99	2	1.5	yes	3.1	yes (1)	0.192		
5	38	F	RRMS	246	20 days	20	3.5	yes	0.86	no		Copaxone, prednisone	Betaferon, Avonex
6	28	F	RRMS	62.5	4	16	1.5	yes	1.77	no		Avonex	Betaferon, IVIG
7	35	F	RRMS	70	30.5	3	0	yes	1.1	yes	0.18	IVIG	
8	29	M	RRMS	31.5	2	5	2	yes	1.09	no	0.733	Avonex	
9	41	F	RRMS	100	37	3	1.5	yes	1.16	no	0.222		
10	48	F	RRMS	243	14	15	3	yes	1.07			Tysabri	Rebif, Copaxone
11	36	M	RRMS	116	7	9	3	yes	0.7	no	0.22	Tysabri	Betaferon
12	44	F	RRMS	112	59	12	2	yes	0.46	no	0.093	Copaxone	Betaferon
13	37	F	RRMS	48.5	1	9	3.5	yes	1.49	no	0.146	IVIG	Betaferon
14	40	F	RRMS	81	3	7	1	yes	1.13	no	0.189	Betaferon	
15	39	M	RRMS	40	3	4	1.5	yes	0.53	no	0.078		
16	37	F	RRMS	49	2		2.5	yes	1.39	no	0.129		
17	22	F	RRMS	56.5	1 day	2	2	yes	1.23	no	0.323		
18	37	M	RRMS	245	23	4	2	yes	0.6	yes (1)	0.09		
19	43	F	RRMS	252.5	9.5	4	2	yes	3.22	no	0.178	Copaxone	Betaferon
20	70	F	RRMS	346	80	5	1.5	yes	1	no			
21	38	F	RRMS	160.5	3	10	2	yes	1.94	yes (1)		Copaxone	Avonex, Betaferon
22	47	F	RRMS	342	8 days	4	2	yes	0.85	no			
23	27	F	RRMS	7	22 days	2	2	yes	0.94	yes (1-2)	0.239		
24	44	F	RRMS	254	11 days	12	1	yes	1.13	yes	0.48	IVIG	Rebif
25	56	F	RRMS	112	63	4	1.5	yes	0.57				
26	38	M	RRMS	115	7	8	1	yes	0.63	no	0.213		
27	30	M	RRMS	109	38.5	2	2	yes	0.45	no	0.048	Copaxone	Avonex
28	45	F	RRMS	194	11	6	3	yes	1.19	no		Tysabri	Rebif, Avonex, IVIG
29	43	M	RRMS	247	7 days	8	2.5	yes	0.73	no			Betaferon
30	37	M	RRMS	17.5	1	2	2	yes	0.66	no	0.116		
31	33	M	RRMS	65	1	9	2	yes	1.14	no	0.476	Copaxone	Betaferon
32	35	M	RRMS	130	76	5	2	yes	0.71	yes (1)	0.086		Copaxone
33	45	F	RRMS	0.25	6 days	1	2	yes	2.89	yes			
34	30	M	RRMS	121				yes	0.66		0.045	Novantrone	Coaxone, Avonex
35	28	F	RRMS	33.5	19	4				no	0.302	Betaferon	IVIG
1	35	M	SPMS	129.5	110	4	6	yes	1.42	yes	0.873	Novantrone, 84 mg/kvm	Avonex, Copaxone
2	63	M	SPMS	341	310.5	4	6	yes	0.53	yes	0.073		Copaxone
3	56	M	SPMS	359.5	17	19	7.5	yes	0.58	yes	0.192		Rebif, Mitoxanotrone
4	67	F	SPMS	266.5	10	6	5	yes	0.95		0.314	Tysabri	Mitoxantron
5	68	M	SPMS	538	336	6	4	yes	0.5	no	0.147		
6	66	M	SPMS	429	162	10	6	yes	0.5	no	0.084		
7	68	F	SPMS	466	128	8	3	yes	0.77	no	0.12		
8	66	F	SPMS	280	254	3	6	yes	0.91	no	0.131		Betaferon
9	68	F	SPMS	246	105	5	3.5	yes	0.53		0.055		
10	56	M	SPMS	175	43	4	6	yes	0.9		0.07		Copaxone, Betaferon
11	56	F	SPMS	206	179	3	5.5	yes	0.63	no			
12	58	F	SPMS	112.5	84	7	6	yes	0.88	yes (1)	0.231		Betaferon, Mitoxantron
13	62	M	SPMS	182	69	2	3.5	yes	0.93	yes (1)	0.127		Rebif
14	57	F	SPMS	261.5	261	1	5	yes	0.82	no	0.334		

ID	Age	Gender	MS-type	Disease duration	Time from latest relapse	No.of relapses	EDSS	OCB -IgG	IgG-index	OCB-IgM	IgM-index	Treatment	Previous treatment
15	53	F	SPMS	309	38	13	6.5	yes	0.49	yes (2-3)	0.352		Mitoxantron
16	59	F	SPMS	300	92	6	6	no	0.61	yes (1)	0.091		
17	66	F	SPMS	315	183	3	3	yes	0.54	no	0.084		
18	62	M	SPMS	145	145	1	6	yes	0.49	no	0.134		
19	66	F	SPMS	353	353	1	6	no	0.49	no	0.077		
